# Association of cardiovascular health with reproductive lifespan and pregnancy loss: insights from NHANES 2005–2018

**DOI:** 10.3389/fendo.2025.1597097

**Published:** 2025-05-26

**Authors:** Yang Yan, Jiajia Chen, Jinlong Qin, Min Yu, Meirong Du

**Affiliations:** ^1^ Laboratory of Reproduction Immunology, Shanghai Key Laboratory of Female Reproductive Endocrine Related Diseases, Obstetrics and Gynecology Hospital, Fudan University Shanghai Medical College, Shanghai, China; ^2^ Department of Obstetrics and Gynecology, Shanghai Fourth People’s Hospital, School of Medicine, Tongji University, Shanghai, China; ^3^ State Key Laboratory of Quality Research in Chinese Medicine and School of Pharmacy, Macau University of Science and Technology, Macau, Macau SAR, China

**Keywords:** cardiovascular health, reproductive lifespan, pregnancy loss, inflammation, NHANES

## Abstract

**Background:**

Altered reproductive timing of females has close relations to long-term health. Since the cardiovascular system delivers oxygen, nutrients, and hormones throughout the body, cardiovascular health (CVH) may significantly impact hormonally controlled events such as pregnancy, menarche, and menopause. This study sought to determine whether CVH is associated with reproductive lifespan and pregnancy loss, and the mediating role of inflammation.

**Methods:**

Female participants (3964) from the National Health and Nutrition Examination Survey (NHANES) 2005–2018 were employed in this cross-sectional investigation. The Life’s Essential 8 (LE8) score was categorized into low (<50), moderate (50–79), and high (≥80) CVH. The years between menarche and menopause age was computed as the reproductive lifespan. Pregnancy loss was determined by the discrepancy between the total number of pregnancies and the number of live births. We conducted multivariable linear regression models and zero-inflated negative binomial regression models to investigate the prospective association of CVH with reproductive lifespan and pregnancy loss while accounting for various potential confounders. Mediation analysis was applied to explore the function of inflammation.

**Results:**

After multivariate adjustment, higher CVH levels were notably associated with lower reproductive lifespan (β=−0.32, 95% CI: −0.47, −0.17, *P*<0.001) and lower number of pregnancy losses (β=−0.04, 95% CI: −0.07, −0.01, *P*=0.012). Specifically, increased CVH levels were associated with increased age at menarche (β=0.14, 95% CI: 0.10, 0.18, *P*<0.001) and decreased age at menopause (β=−0.18, 95% CI: −0.33, −0.04, *P*=0.014). Furthermore, a linear correlation was observed between CVH and reproductive lifespan (*P*<0.001), while the number of pregnancy losses decreased as CVH levels increase within a certain range and approximately presented an L-shaped relationship (*P*=0.009). Subgroup analyses proved a stronger inverse association between CVH and reproductive lifespan among never-married women (*P* for interaction<0.001), whereas no significant interaction existed between CVH and pregnancy loss. Inflammation biomarker alkaline phosphatase (ALP) mediated 9.4% of the association between CVH and reproductive lifespan (*P*=0.048).

**Conclusions:**

Higher CVH levels were associated with shorter reproductive lifespan and lower prevalence of pregnancy loss at population level, and inflammation may mediate the association of CVH with reproductive lifespan. Comprehensive management of CVH in women may be vital to safeguard their reproductive health.

## 1 Introduction

Pregnancy, menarche, and menopause are all key events of female reproductive life and are heavily influenced by hormones. The reproductive lifespan spans from the first to the last menstrual cycle of women (1), while pregnancy loss is known as the discrepancy between the tally of confirmed pregnancies and live newborns, which primarily involves adverse pregnancy events, such as miscarriages, stillbirths, and genetic terminations (2). Since hormone levels and cardiovascular health (CVH) have a complicated relationship, exposure to multiple factors that impair CVH during critical windows could alter the reproductive lifespan and pregnancy outcome. Growing evidence shows that cardiovascular diseases (CVDs) interfere with female reproductive factors, including fertility. On one hand, women with cardiovascular risk factors may heighten the risk of adverse perinatal outcomes during pregnancy (3, 4), and pregnant individuals with preexisting CVDs have elevated incidence rates of preeclampsia, placental abruption, and postpartum hemorrhage (5, 6). On the other hand, CVDs can cause endocrine issues and then alter ovulation and the menstrual cycle (7, 8), ultimately affecting fertility. Hence, the impact of CVH on reproductive health could be substantial.

More recently, the American Heart Association (AHA) introduced an emerging CVH indicator, termed as Life’s Essential 8 (LE8) score (9), which consists of diet, physical activity, smoking, sleep health, body mass index (BMI), untreated total cholesterol, fasting blood glucose, and untreated blood pressure (BP). LE8 was a redefined and recalculated indicator based on Life’s Simple 7, which integrates the above metrics without the sleep domain. It has been demonstrated that sleep quality is associated with female reproductive factors (10). LE8 underpins our study since it reflects up-to-date clinical guidelines and more appropriate responses to interindividual differences and changes. Higher CVH scores indicate better health and lower risk for multiple subsequent cardiovascular and non-cardiovascular events.

Inflammation responses are implicated in the physiological and pathological mechanisms of female reproductive events (11). Alkaline phosphatase (ALP) is an enzyme with multifaceted anti-inflammatory action, which mainly presents in the bone and liver, as well as the placenta, intestine, kidney, and leukocytes (12, 13). Increased serum ALP activity demonstrates significant covariation with inflammation (14, 15) and CVDs (16). In addition, detecting markedly high levels of ALP during the pregnancy might indicate additional risk for adverse obstetrics and perinatal outcome such as preterm labor, gestational diabetes, and hypertensive disorders (17, 18). Of note, it remains to be elucidated as to whether the immune response modulated through the ALP pathway could play important roles in the relationship between CVH and female reproductive health, which could promote a deeper understanding of the mechanistic role orchestrated by ALP in immune metabolism.

The National Health and Nutrition Examination Survey (NHANES) is an ethically approved, cross-sectional, nationwide survey that tracks Americans’ health and nutritional status over the past few decades. Drawing on diverse NHANES cycles, the associations of CVH with life expectancy, metabolic dysfunction, female infertility, cancer mortality, and liver, lung, renal, psychiatry diseases have been highlighted (19–23). However, the association of CVH with reproductive lifespan and pregnancy loss remains poorly understood in the general population. Herein, we comprehensively evaluated the above associations in a nationally representative sample of U.S. women from the 2005–2018 NHANES and sought to identify the mediating effects of immune response involving the ALP pathway.

## 2 Materials and methods

### 2.1 Study population

**Figure 1:**
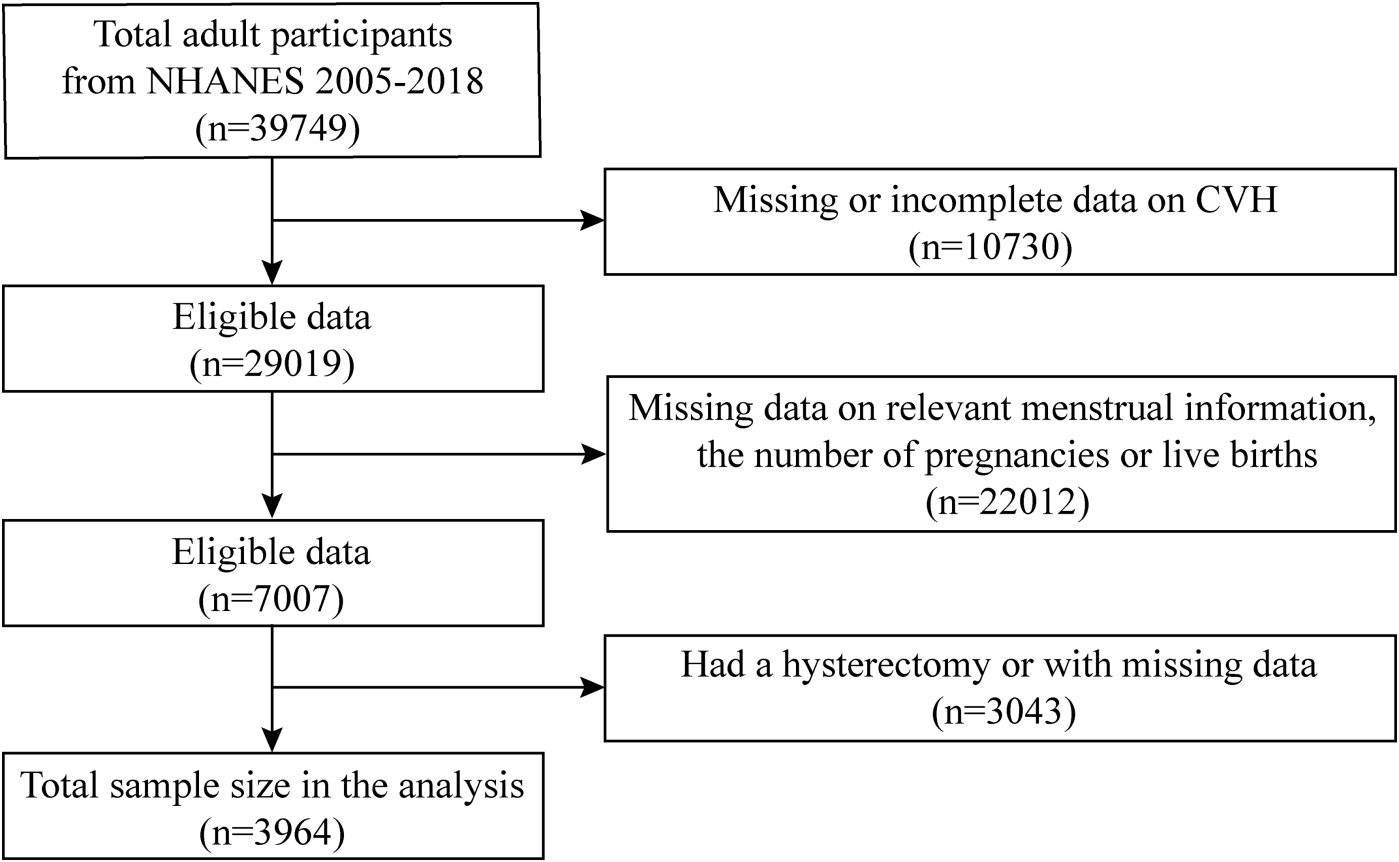
Flow chart for participant recruitment from NHANES 2005–2018.

The NHANES is conducted by the National Center for Health Statistics at the Centers for Disease Control and Prevention. It is based on a multilevel probability sampling design for interviews, examinations, and laboratory items. Our data were collected from the 2005–2018 NHANES spanning seven consecutive waves. In total, 39,749 participants were included, and our analyses were limited to 3964 participants eventually. We only included female participants aged 40 and above who reported their age at menarche and natural menopause in the reproductive health questionnaire. Exclusions were implemented for the following individuals (1): missing or incomplete data required for assessing CVH (n=10,730), (2) missing data on relevant menstrual information and the number of pregnancies or live births (n=22,012), and (3) had a hysterectomy or with missing data (n=3043) (**Figure 1**).

### 2.2 Definitions of LE8 and CVH evaluation

The CVH status was quantified by the LE8 score, which was just launched in 2022; the scoring algorithm incorporates four health behaviors including diet, physical activity, tobacco/nicotine exposure, and sleep, while four health factors include BMI, non-high-density lipoprotein cholesterol, blood glucose, and BP (9). Scoring standards range from 0 to 100 for each indicator. Details on the LE8 CVH score calculation for each metrics have been previously published and are provided in **Supplementary Table 1** (9, 24). The LE8 CVH score was computed as the average of these eight metrics and was divided into poor (0–49), moderate (50–79), and high (80–100) groups (4, 9).

Diet metric was evaluated by the Dietary Approaches to Stop Hypertension (DASH) diet score. Information about physical activity times, tobacco/nicotine use, sleep durations, history of diabetes, and medication was collected from self-report questionnaires. During the physical examination, measurements were taken of height, weight, and BP. BMI was computed by weight (kg) divided by height squared (m^2^). The average of the first three diastolic and systolic BP readings was recorded. Laboratory analysis of blood samples was performed to quantify non-high-density lipoprotein cholesterol, fasting glucose, and glycated hemoglobin levels.

### 2.3 Reproductive endpoints assessment

To calculate the length of the reproductive lifespan, we collected the self-reported information on age at the first and the last menstrual periods from the Reproductive Health Questionnaire, relying on answers to the questions “How old were you when you had your first menstrual period?” and “About how old were you when you had your last menstrual period?”, respectively. Reproductive lifespan was further analyzed as a continuous variable.

### 2.4 Pregnancy loss assessment

Pregnancy loss was appraised basing on self-reported reproductive health outcomes obtained through computer-assisted personal interviews and was viewed as a continuous variable for analysis. The number of initiated pregnancies was determined by responses to the following question: “How many times have you been pregnant? Be sure to count all your pregnancies including current pregnancy, live births, miscarriages, stillbirths, tubal pregnancies, and other pregnancies.” For assessing the number of deliveries with live newborns, participants were asked “How many of your deliveries resulted in a live birth? Count the number of total deliveries, not number of live born children.” Pregnancy loss was documented when there was at least one more pregnancy than live birth.

### 2.5 Covariates and mediators

Drawing from previous research findings and clinical insights, the following demographic and socioeconomic covariates that had been proven to be correlated with cardiovascular health and reproductive factors were included: age, race/ethnicity (non-Hispanic white, non-Hispanic black, Mexican American, or others), education level (less than high school, high school, or more than high school), poverty income ratio (PIR), and marital status (married/living with a partner, widowed/divorced/separated, or never married), all of which were based on self-reported information.

As for the effect modifier variable, serum ALP was employed as a continuous variable, which has been widely utilized in NHANES studies for measuring inflammation. The variable name “LBXSAPSI” in the NHANES laboratory dataset was used to test ALP serum levels. Serum ALP activity was quantified using the Beckman Synchron LX20 Analyzer (NHANES 2005–2006), the Beckman Synchron LX20 Analyzer and the Beckman Coulter DxC800 Synchron Clinical System (NHANES 2007–2008), the Beckman Coulter DxC800 Synchron Clinical System (NHANES 2009–2014), the Beckman Coulter DxC800 and DxC660i Synchron Clinical Systems (NHANES 2015–2016), and the Roche Cobas 6000 Chemistry Analyzer (NHANES 2017–2018). Comprehensive data regarding the mentioned variables are obtainable through the NHANES database (https://www.cdc.gov/nchs/nhanes).

### 2.6 Statistical analysis

Categorical variables are presented as percentages, while continuous variables are indicated as mean ± standard deviation. All regression models were weighted using the complex survey design of NHANES. Specifically, we used the fasting subsample weights (WTSAF2YR) because several variables, including serum alkaline phosphatase (ALP) and fasting glucose, were measured in the fasting subsample. Stratification (SDMVSTRA), clustering (SDMVPSU), and appropriate weights were incorporated into all analyses following NHANES analytic guidelines.

In order to determine the association of CVH with reproductive lifespan and pregnancy loss, we fitted three models with increasing covariate adjustments to account for the potential confounding impact of different covariables: unadjusted (Model 1); adjusted for age and race/ethnicity (Model 2); adjusted for age, race/ethnicity, education level, PIR, and marital status (Model 3). Reproductive lifespan, treated as a continuous outcome, was analyzed using survey-weighted multivariable linear regression models. For pregnancy loss, which is a count variable with excess zeros, we also employed zero-inflated negative binomial (ZINB) regression models to appropriately model its distribution and obtain valid estimates. Further, a restricted cubic spline (RCS) regression model, using four knots placed at the 5th, 35th, 65th, and 95th percentiles of the CVH score distribution, was constructed to evaluate the non-linear relationship between CVH and reproductive lifespan and pregnancy loss, respectively. Subgroup analyses were performed by race/ethnicity, age, education level, PIR, and marital status. At last, the R package mediation (version 4.5) was used for mediation analyses, examining whether inflammatory mediated the association of CVH with reproductive lifespan and pregnancy loss. All above statistical examinations were performed in R (version 4.3.3) and EmpowerStats (version 4.2). A *P* value was deemed statistically significant if it was 0.05 or less.

## 3 Results

### 3.1 Baseline characteristics of participants

The general characteristics of the selected participants stratified by CVH levels were summarized in **Table 1**. In total, 3964 female adults were included in this study, in which 984, 2542, and 438 individuals exhibited low (LE8<50), moderate (50≤LE8<80), and high (LE8≥80) CVH, respectively. Overall, women characterized by higher CVH levels were more frequently to be younger, non-Hispanic white, and married/living with a partner. Moreover, they possessed higher educational levels and PIR but lower number of pregnancies and live births. Additionally, the average reproductive lifespan was 34.86 ± 7.63 years, and the average number of pregnancy losses was 0.74 ± 1.26 in the overall enrolled population. The average total CVH score was 60.70 ± 14.82, while it was 41.83 ± 6.43 in low CVH grade, 63.66 ± 8.15 in moderate CVH grade, and 85.90 ± 4.71 in high CVH grade, respectively. Detailed significant differences of eight LE8 metric scores were also observed among three CVH grades.

**Table 1:** Baseline characteristics of participants with different CVH levels estimated from LE8 score.

Characteristics	Total	Low (LE8 < 50)	Moderate (50 ≤ LE8 < 80)	High (LE8 ≥ 80)	*P* value
No. of participants in sample	3964	984	2542	438	
Age, y (SD)	61.82 ± 11.80	62.25 ± 10.36	62.32 ± 11.99	57.91 ± 12.95	< 0.001
Age at menarche, y (SD)	12.88 ± 1.80	12.71 ± 1.84	12.90 ± 1.78	13.13 ± 1.74	< 0.001
Age at menopause, y (SD)	47.73 ± 7.47	47.70 ± 6.86	47.84 ± 7.44	47.21 ± 8.81	0.157
Reproductive lifespan, y (SD)	34.86 ± 7.63	34.99 ± 7.13	34.94 ± 7.56	34.09 ± 8.99	0.681
Number of pregnancies, n (SD)	3.65 ± 2.24	4.02 ± 2.49	3.62 ± 2.19	3.01 ± 1.64	< 0.001
Number of live births, n (SD)	2.98 ± 1.84	3.26 ± 2.06	2.96 ± 1.79	2.42 ± 1.27	< 0.001
Number of pregnancy losses, n (SD)	0.74 ± 1.26	0.82 ± 1.31	0.72 ± 1.25	0.70 ± 1.12	0.207
PIR (SD)	2.56 ± 1.63	1.93 ± 1.38	2.61 ± 1.62	3.66 ± 1.57	< 0.001
Race/ethnicity, n (%)					< 0.001
Non-Hispanic White	1865 (47.05%)	417 (42.38%)	1187 (46.70%)	261 (59.59%)	
Non-Hispanic Black	712 (17.96%)	241 (24.49%)	432 (16.99%)	39 (8.90%)	
Mexican American	592 (14.93%)	180 (18.29%)	376 (14.79%)	36 (8.22%)	
Others	795 (20.06%)	146 (14.84%)	547 (21.52%)	102 (23.29%)	
Education level, n (%)					< 0.001
Less than high school	1030 (25.98%)	347 (35.26%)	659 (25.92%)	24 (5.48%)	
High school	943 (23.79%)	268 (27.24%)	616 (24.23%)	59 (13.47%)	
More than high school	1991 (50.23%)	369 (37.50%)	1267 (49.84%)	355 (81.05%)	
Marital status, n (%)					< 0.001
Married/Living with a partner	2010 (50.71%)	422 (42.89%)	1288 (50.67%)	300 (68.49%)	
Widowed/Divorced/Separated	1590 (40.11%)	464 (47.15%)	1024 (40.28%)	102 (23.29%)	
Never married	364 (9.18%)	98 (9.96%)	230 (9.05%)	36 (8.22%)	
AHA LE8 score (SD)					
Total CVH score	60.70 ± 14.82	41.83 ± 6.43	63.66 ± 8.15	85.90 ± 4.71	< 0.001
Mean DASH diet score	50.53 ± 32.56	29.72 ± 27.57	54.12 ± 31.04	76.47 ± 24.11	< 0.001
Mean physical activity score	36.21 ± 44.47	7.05 ± 22.02	38.05 ± 44.35	91.05 ± 21.32	< 0.001
Mean tobacco/nicotine exposure score	76.79 ± 33.13	58.42 ± 39.51	81.38 ± 29.47	91.42 ± 17.11	< 0.001
Mean sleep health score	81.90 ± 25.14	70.13 ± 29.44	84.38 ± 23.16	93.95 ± 13.07	< 0.001
Mean BMI score	57.19 ± 34.60	34.67 ± 30.42	60.52 ± 32.71	88.50 ± 19.13	< 0.001
Mean blood lipid score	58.31 ± 30.27	44.27 ± 29.81	60.96 ± 29.11	74.47 ± 25.30	< 0.001
Mean blood glucose score	72.56 ± 27.99	55.51 ± 29.03	75.77 ± 25.68	92.19 ± 16.56	< 0.001
Mean BP score	52.11 ± 33.41	34.84 ± 28.55	54.14 ± 32.51	79.17 ± 26.69	< 0.001

Mean (SD) for continuous variables: the *P* value was calculated by the weighted linear regression model.

Percentages for categorical variables: the *P* value was calculated by the weighted chi-square test.

CVH, cardiovascular health; LE8, Life’s Essential 8; SD, standard deviation; PIR, poverty income ratio; AHA, American Heart Association; DASH, Dietary Approaches to Stop Hypertension; BMI, body mass index; BP, blood pressure.

### 3.2 Association of CVH with reproductive lifespan

**Table 2** displays the association between CVH and reproductive lifespan grounded by the multivariable linear regression analysis. Our findings indicated that higher CVH levels were linked to lower reproductive lifespan in the unadjusted and fully adjusted models, whereas it was not significant in the minimally adjusted model. As shown, when full adjustments were made, it was observed that for every one-unit increment in the continuous CVH score, there was a corresponding decrease of 0.32 years in reproductive lifespan (β=−0.32, 95% CI: −0.47, −0.17, *P*<0.001). Contrasted with the lowest CVH level, individuals with the moderate and highest CVH levels exhibited decreased reproductive lifespan, with reductions of 0.65 years and 1.15 years (β=−0.65, 95% CI: −1.14, −0.15, *P*=0.010; β=−1.15, 95% CI: −1.94, −0.36, *P*=0.004; *P* for trend=0.002), respectively.

**Table 2:** The association between the LE8 CVH score and reproductive lifespan.

CVH metrics	Model 1 [β (95% CI) *P*]	Model 2 [β (95% CI) *P*]	Model 3 [β (95% CI) *P*]
Total CVH score (per 10 scores)	−0.18 (−0.34, −0.02) 0.024	0.04 (−0.11, 0.18) 0.594	−0.32 (−0.47, −0.17) < 0.001
CVH categories			
Low (LE8 < 50)	Ref.	Ref.	Ref.
Moderate (50 ≤ LE8 < 80)	−0.05 (−0.61, 0.51) 0.856	−0.08 (−0.58, 0.41) 0.744	−0.65 (−1.14, −0.15) 0.010
High (LE8 ≥ 80)	−0.90 (−1.76, −0.04) 0.040	0.45 (−0.33, 1.22) 0.258	−1.15 (−1.94, −0.36) 0.004
*P* for trend	0.137	0.326	0.002

Model 1 was unadjusted for covariates; Model 2 enhanced Model 1 by including age and race/ethnicity; Model 3 further augmented Model 2 by integrating education level, PIR, and marital status.

CVH, cardiovascular health; LE8, Life’s Essential 8.

**Figure 2:**
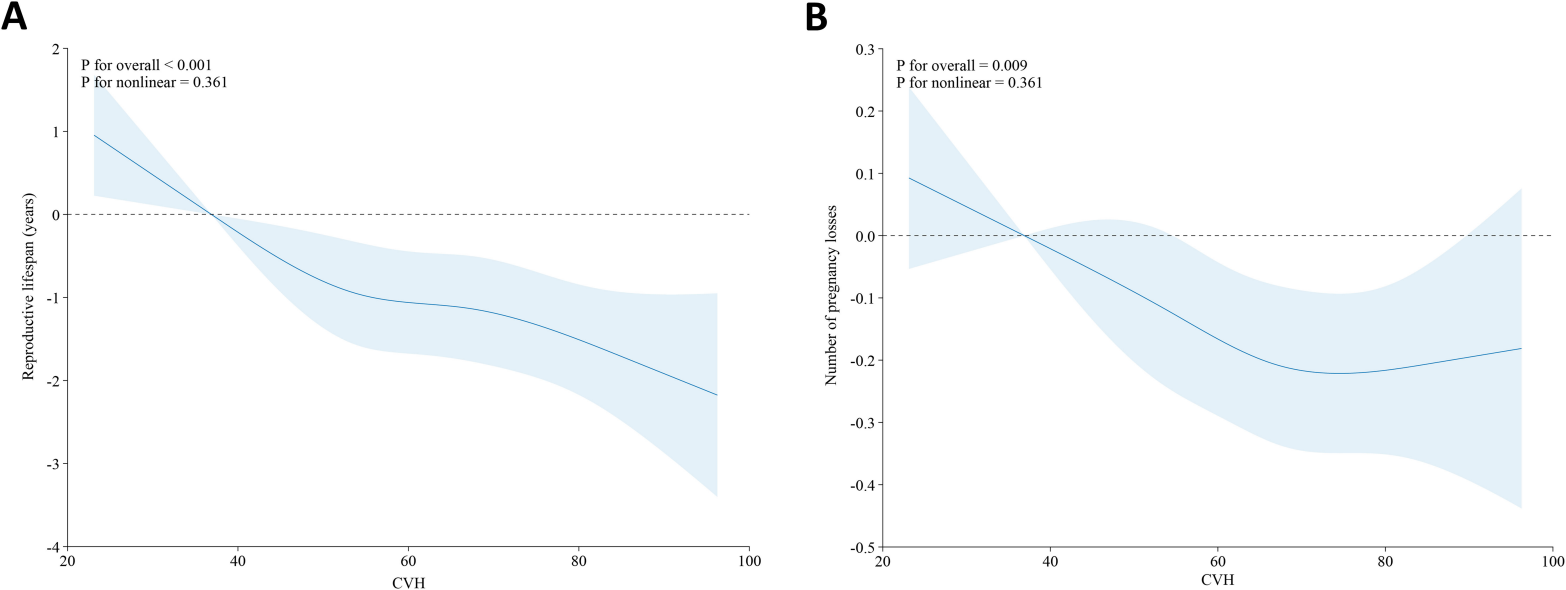
Association between CVH and reproductive lifespan **(A)** and pregnancy loss **(B)** in the U.S. female adults based on the RCS models. Adjusted: age, race/ethnicity, education level, PIR, and marital status. The solid blue line and the shaded area denote the estimated β values and their corresponding 95% CIs, respectively. CVH, cardiovascular health; RCS, restricted cubic spline; PIR, poverty income ratio.

By using the RCS model with all confounders fully adjusted, we further found that there was a linear linkage between CVH and reproductive lifespan (*P* for overall<0.001, *P* for nonlinear=0.361), as shown in **Figure 2A**. In other words, the reproductive lifespan of women diminishes with increasing CVH levels.

More specifically, increased CVH levels were associated with increased age at menarche and decreased age at menopause. As presented in **Table 3**, higher CVH levels were linked to higher age at menarche, both in the unadjusted model and the minimally/fully adjusted model. For every one-unit increment in the continuous CVH score, there was a corresponding increase of 0.14 years in the age at menarche after adjusting for all the proposed confounders (β=0.14, 95% CI: 0.10, 0.18, *P*<0.001). In addition, participants with the moderate and highest CVH levels exhibited higher age at menarche compared to the lowest CVH level (β=0.23, 95% CI: 0.10, 0.36, *P*<0.001; β=0.64, 95% CI: 0.42, 0.85, *P*<0.001; *P* for trend<0.001). Whereas the age at menopause dropped as CVH levels improved when confounders were fully adjusted (β=−0.18, 95% CI: −0.33, −0.04, *P*=0.014), but this association ceased to be significant after being fully adjusted in terms of a categorical CVH variable.

**Table 3:** The association of the LE8 CVH score with age at menarche and age at menopause.

CVH metrics	Age at menarche (years)			Age at menopause (years)		
	Model 1 [β (95% CI) *P*]	Model 2 [β (95% CI) *P*]	Model 3 [β (95% CI) *P*]	Model 1 [β (95% CI) *P*]	Model 2 [β (95% CI) *P*]	Model 3 [β (95% CI) *P*]
Total CVH score (per 10 scores)	0.09 (0.05, 0.13) < 0.001	0.10 (0.06, 0.14) < 0.001	0.14 (0.10, 0.18) < 0.001	−0.10 (−0.25, 0.06) 0.231	0.14 (0.00, 0.28) 0.046	−0.18 (−0.33, −0.04) 0.014
CVH categories						
Low (LE8 < 50)	Ref.	Ref.	Ref.	Ref.	Ref.	Ref.
Moderate (50 ≤ LE8 < 80)	0.19 (0.06, 0.32) 0.005	0.18 (0.05, 0.31) 0.008	0.23 (0.10, 0.36) < 0.001	0.14 (−0.41, 0.69) 0.624	0.10 (−0.38, 0.57) 0.695	−0.42 (−0.89, 0.05) 0.083
High (LE8 ≥ 80)	0.42 (0.21, 0.62) < 0.001	0.49 (0.28, 0.69) < 0.001	0.64 (0.42, 0.85) < 0.001	−0.49 (−1.33, 0.36) 0.258	0.93 (0.20, 1.67) 0.013	−0.52 (−1.27, 0.24) 0.183
*P* for trend	< 0.001	< 0.001	< 0.001	0.646	0.018	0.149

Model 1 was unadjusted for covariates; Model 2 enhanced Model 1 by including age and race/ethnicity; Model 3 further augmented Model 2 by integrating education level, PIR, and marital status.

CVH, cardiovascular health; LE8, Life’s Essential 8.

### 3.3 Association of CVH with pregnancy loss

To understand the relationship between CVH and pregnancy loss, we still fitted three models with increasing covariate adjustments (**Table 4**). Both the unadjusted model and the minimally/fully adjusted model revealed that higher CVH levels were tied to lower risk of pregnancy loss. For every one-unit increment in the continuous CVH score, there was a corresponding decline of 0.04 times in the number of pregnancy losses (Model 1: β=−0.04, 95% CI: −0.07, −0.01, *P*=0.003; Model 2: β=−0.04, 95% CI: −0.07, −0.01, *P*=0.007; Model 3: β=−0.04, 95% CI: −0.07, −0.01, *P*=0.012). As a categorical variable, participants with the moderate and highest CVH levels exhibited lower risk of pregnancy loss in contrast to the lowest CVH level (all *P* for trend<0.05). In ZINB regression models (**Supplementary Table 2**), higher CVH score was significantly associated with fewer pregnancy losses, and the associations remained consistent across all models. After adjusting for age and socioeconomic factors, each one-unit increase in CVH score was associated with a 0.08 decrease in the expected number of pregnancy losses (β=−0.08, 95% CI: −0.13, −0.02, *P*=0.005).

**Table 4:** The association between the LE8 CVH score and pregnancy loss.

CVH metrics	Model 1 [β (95% CI) *P*]	Model 2 [β (95% CI) *P*]	Model 3 [β (95% CI) *P*]
Total CVH score (per 10 scores)	−0.04 (−0.07, −0.01) 0.003	−0.04 (−0.07, −0.01) 0.007	−0.04 (−0.07, −0.01) 0.012
CVH categories			
Low (LE8 < 50)	Ref.	Ref.	Ref.
Moderate (50 ≤ LE8 < 80)	−0.10 (−0.20, −0.01) 0.038	−0.09 (−0.19, 0.01) 0.065	−0.08 (−0.18, 0.02) 0.104
High (LE8 ≥ 80)	−0.13 (−0.28, 0.03) 0.108	−0.11 (−0.27, 0.04) 0.152	−0.10 (−0.27, 0.06) 0.219
*P* for trend	0.017	0.033	0.046

Model 1 was unadjusted for covariates; Model 2 enhanced Model 1 by including age and race/ethnicity; Model 3 further augmented Model 2 by integrating education level, PIR, and marital status.

CVH, cardiovascular health; LE8, Life’s Essential 8.

We further employed the RCS analysis to explore the potential non-linear association of CVH with pregnancy loss. As shown in **Figure 2B**, CVH levels and the number of pregnancy losses approximate an L-shaped relationship (*P* for overall=0.009, *P* for nonlinear=0.361), which means the number of pregnancy losses decreases as CVH levels increase within a certain range.

### 3.4 Subgroup analysis

**Figure 3:**
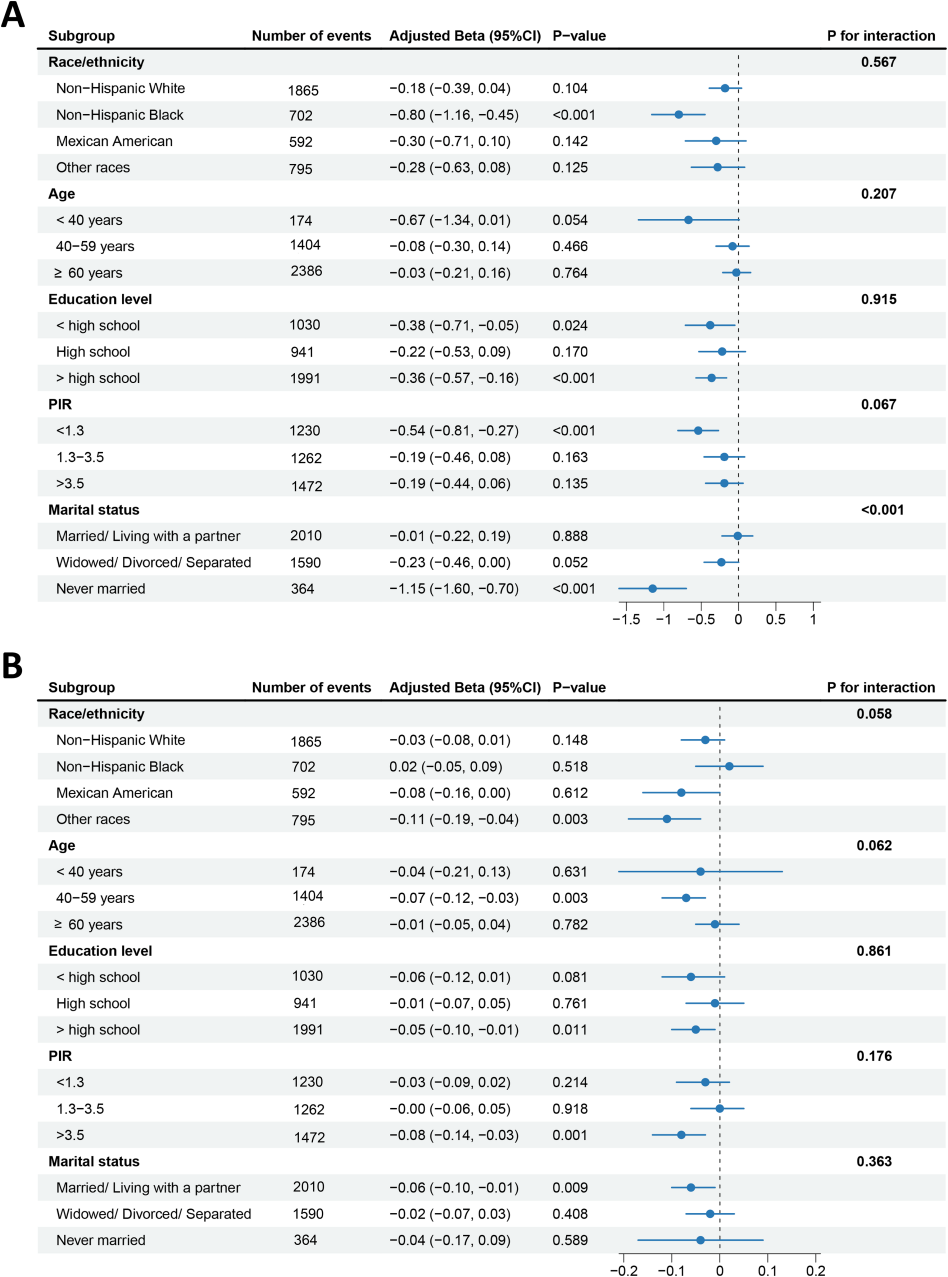
Forest plots of subgroup analyses of CVH with reproductive lifespan **(A)** and pregnancy loss **(B)**. Adjusted: race/ethnicity, age, education level, PIR, and marital status, except the stratified variable itself. CVH, cardiovascular health; PIR, poverty income ratio.

To further assess the resilience of results, we stratified the data by race/ethnicity, age, education level, PIR, and marital status. CVH levels presented a more pronounced negative correlation of reproductive lifespan in postmenopausal women of non-Hispanic black, less than or more than high school, PIR<1.3, and never married (**Figure 3A**), and reproductive lifespan values were decreased by 0.8 years (β=-0.80, 95% CI: −1.16, −0.45, *P*<0.001), 0.38 years (β=−0.38, 95% CI: −0.71, −0.05, *P*=0.024), 0.36 years (β=−0.36, 95% CI: −0.57, −0.16, *P*<0.001), 0.54 years (β=−0.54, 95% CI: −0.81, −0.27, *P*<0.001), and 1.15 years (β=−1.15, 95% CI: −1.60, −0.70, *P*<0.001) with each unit increase in the continuous CVH score, respectively. Moreover, we observed a differential relationship of CVH with reproductive lifespan by marital status, with this negative relationship more pronounced among never-married women (*P* for interaction<0.001), whereas there was no significant interaction of other stratified variables between CVH levels and reproductive lifespan.

In addition, as CVH levels increase, the frequency of pregnancy losses is lower for women of other races, middle-age (40–59 years old), more than high school education level, PIR>3.5, and married/living with a partner (**Figure 3B**), with the prevalence of pregnancy losses decreased by 0.11 times (β=−0.11, 95% CI: −0.19, −0.04, *P*=0.003), 0.07 times (β=−0.07, 95% CI: −0.12, −0.03, *P*=0.003), 0.05 times (β=−0.05, 95% CI: −0.10, −0.01, *P*=0.011), 0.08 times (β=−0.08, 95% CI: −0.14, −0.03, *P*=0.001), and 0.06 times (β=−0.06, 95% CI: −0.10, −0.01, *P*=0.009) with each unit increase in the continuous CVH score, respectively. However, no significant interaction was noted between CVH levels and pregnancy loss (all *P* for interaction>0.05).

### 3.5 Inflammation mediated the association between CVH and reproductive lifespan

**Figure 4:**
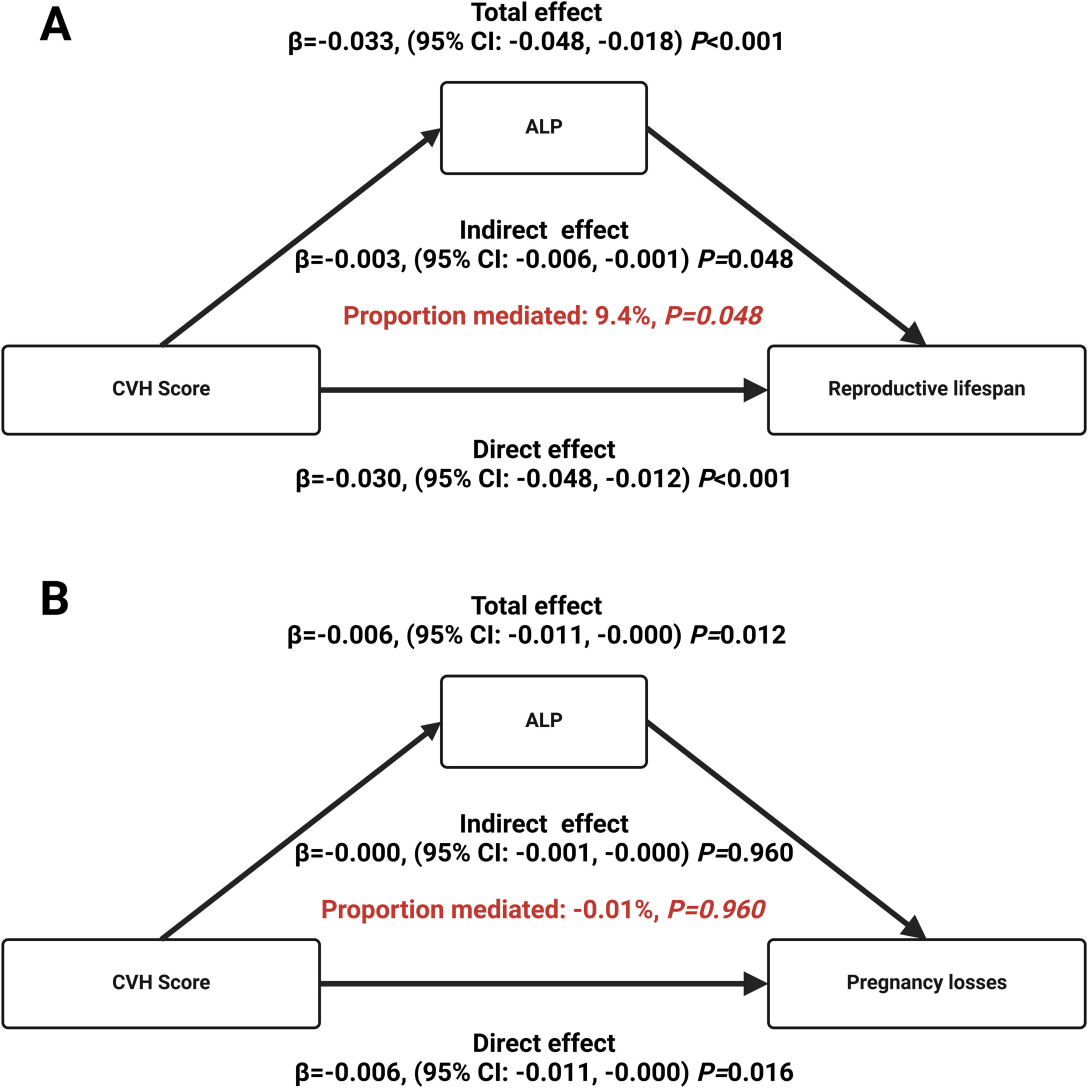
Mediation effects of inflammation biomarker ALP in the associations of CVH with reproductive lifespan **(A)** and pregnancy loss **(B)**. CVH, cardiovascular health; ALP, alkaline phosphatase.

The current study estimates the mediating effect of inflammation biomarker ALP, based on the hypothesis that CVH has influence on reproductive lifespan through ALP. As shown in **Figure 4**, ALP level mediated the negative association between CVH and reproductive lifespan, accounting for 9.4% (*P* value for mediation=0.048) of the effect (**Figure 4A**). In terms of the association between CVH and pregnancy loss (**Figure 4B**), ALP level mediated significant direct effect, but the indirect effect was not significant (*P* value for mediation>0.05).

## 4 Discussion

In a representative sample of U.S. postmenopausal women from NHANES 2005–2018, adherence to the LE8 metrics for CVH was associated with a shorter reproductive lifespan. Notably, the inverse association between CVH and reproductive lifespan was stronger among never-married women. Mediation analyses revealed that inflammation biomarker ALP may mediate the association between CVH and reproductive lifespan. There was compelling evidence indicating that the likelihood of reporting an elevated frequency of pregnancy loss was also lower as CVH levels improve. Further analyses using RCS models revealed a linear relationship between higher CVH levels and shorter reproductive lifespan, while the number of pregnancy losses decreases as CVH levels increase within a specific range and roughly presented an L-shaped relationship.

Reproductive lifespan holds significance of health status and physical aging in women. Since the timing of both onset and cessation of menstruation are known to be independent and vary among individuals, there may be distinct determinants, from hereditary and developmental factors to the combined effects of complex hormonal, environmental, and lifestyle exposures, influencing the reproductive timing (25–28). Thus far, no previous studies have considered the association of CVH estimated from LE8 score with reproductive lifespan, although better CVH was reported to be associated with lower occurrence of female infertility (22, 29). In our large population made of postmenopausal women, higher CVH levels were associated with shorter reproductive lifespan, exactly, delayed menarche and early menopause. It is known that significant overlap existed between female reproductive disorders like infertility, polycystic ovary syndrome, and the risk factors of CVDs as well as metabolic dysfunction (30). Previous pieces of evidence showed that female infertility was related to increased risk of early menopause (31), and the predicted reproductive lifespan of polycystic ovary syndrome cases was extended by an average of two years compared with normo-ovulatory females (32). Rather, in a Mendelian randomization analysis, biomarkers of CVD risk factors as measured by several types of lipids and blood pressure showed no impact on the risk of female infertility; of note, circulating levels of cardiometabolic risk determinants were not available in this investigation (33). The direct comparison of the above studies may be difficult due to different categories of CVH assessments. Notably, we employed LE8 as a novel CVH measurement which considers a broader range of health components. Numerous studies have emphasized the advantages of LE8 as a predictor of cardiovascular-related events. For example, LE8 surpassed LS7 in the prediction of coronary artery stenosis (34), and LE8 showed superior predictive ability for the risk of subsequent CVD mortality in individuals diagnosed with chronic kidney disease (35). Moreover, it is well recognized that sleep quality and pattern are frequently interrelated with reproductive events. Accordingly, the present results which has been disclosed under this newly developed scoring method were likely to be more fully and accurate.

Currently, there is an expanding body of data demonstrating that worse maternal CVH is associated with the incidence of adverse pregnancy outcomes, such as fetal death, preterm birth, preeclampsia, and small-for-gestational age birth (36, 37). Our findings are in agreement with the above evidence, showing that lower degree of CVH was suggestively related to increased number of pregnancy losses, which occur in 12%–15% of pregnancies. Several individual CVH metrics potentially correlated with pregnancy loss have been proven in previous research studies; for example, healthy dietary pattern and moderate occupational physical activity could reduce the chance of pregnancy loss (38, 39), while sedentary behavior, smoking, sleep–wake disorders, overweight or obesity, low-density lipoprotein cholesterol, blood glucose abnormality, and hypertensive disorders are highly associated with pregnancy loss (40–45). Results from the above observational studies may be distorted owing to residual confounding. Herein, LE8 score, which explores the overall effects of numerous health-related factors on CVH, was utilized, thus providing more solid evidence for understanding the diverse determinants affecting pregnancy loss before formulating effective intervention strategies.

While *a priori*, a shorter reproductive lifespan will reduce the chances of initiating pregnancy, there is substantial evidence supporting that extending the length of reproductive lifespan does not necessarily mean better pregnancy outcomes; for example, diabetes, hypertension, and other age-related diseases can negatively impact reproductive endpoints (46–48). Moreover, both premature and delayed timing of menarche and menopause have been correlated with non-reproductive detrimental health outcomes. It should be noted that women experiencing premature menarche or delayed menopause are at higher risk of steroid hormone-driven cancers, like breast, endometrial, and ovarian cancer (49–51). In addition, premature menarche also has been related with premature death, depression, CVDs, obesity, and type 2 diabetes mellitus (52–54), while diminished bone mineral density and a rising incidence of depressive disorders have been linked to delayed menarche (55, 56). Meanwhile, early menopause may increase the likelihood of long-term health issues, including reduced overall survival and increased risk of CVDs, osteoporosis, and mortality (57–59). Our results in the current research align with the above hypothesis that a prolonged reproductive lifespan does not seem to be related to optimal pregnancy outcome, but higher frequency of pregnancy loss. Potential mechanisms of the capacity damage of pregnancy maintenance could be through an increase in maternal age, which may lead to an increase in the rate of aneuploidy in embryos or a higher risk of neuroendocrine disorder, both in the reproductive organs and the entire body system. Given the adverse health effects of extended reproductive lifespan, public health interventions and effective and safe individualized plans could be urgently warranted.

Recent studies have elucidated novel insights into the mechanistic function of ALP in immunometabolic regulations. The potential anti-inflammatory ALP pathways may include the anti-inflammatory adenosine production, lipopolysaccharide detoxification, autophagy-dependent cellular degradation, mitochondrial biogenesis, and caveolin-dependent endocytosis (60–62). In this study, we found that ALP functioned as a critical modifier in the stable negative association between CVH and the duration of reproductive lifespan, accounting for 9.4% of the proportion using mediation analysis. Thus, we speculated that exposure to CVH metrics can balance inflammation states in the organ systems, at least partially, and then potentially lead to the alteration of reproductive events. The well-established mechanisms of immune response induced by the CVH metrics have yet to be determined, but in theory, it can be inferred from the aforementioned mechanism. Apart from the most prominent clinical diagnostic values on hepatobiliary system and musculoskeletal diseases, ALP also experiences noteworthy changes in various types of pathological pregnancy. Current studies have demonstrated that women with habitual abortion exhibit significantly elevated ALP levels compared to control groups (63), and ALP heightens the risk of spontaneous pregnancy loss in pregnant women (64). However, the pathology linking ALP to pregnancy loss is not well established in our cohort. To our knowledge, ALP is present in many tissues in the body while it was only measured in the serum in NHANES; thus, we were unable to confirm the origin of the enzyme, which means that this viewpoint remains speculative, and future researches should focus on specific ALP isozymes for further characterization.

The strength of our study lies in the statistically rigorous sampling design and weighted analysis of the large-scale NHANES data, which enable our findings to be extended to the U.S. general population, although it may not be directly generalized to other study settings or populations. More importantly, previous research has also reported the adverse effect of CVDs on female reproduction, but this is the first effort to focus on the association of CVH with reproductive lifespan and pregnancy loss. The employment of LE8, a currently updated measure of CVH with superior predictive ability, strengthened the credibility and accuracy of the results. The extensive adjustments for confounding factors, including age, race/ethnicity, education level, PIR, and marital status, ensured the validity and stability of the conclusion. There are also some limitations to note. Firstly, our conclusions must be interpreted cautiously in light of the cross-sectional design, which means it is not possible to infer causality. Although they have not always been consistent, few studies reported that reproductive factors such as the number of pregnancies may have influence on the risk of CVDs, supporting a likely possibility of a mutual influence between CVH and reproductive events. Secondly, despite considerably adjusting potential confounders and conducting sensitivity analyses, we were unable to assess the impact of residual confounding factors such as menstrual regularity, which was not available in NHANES. To clarify the underlying mediation effect, more unmeasured confounders need to be accurately identified and deeply evaluated by further prospective studies. Finally, we had difficulties in discarding some misclassified data due to the age information on menstruation. In addition, the calculated number of pregnancy losses was based on self-reports and may be prone to reporting bias.

## 5 Conclusions

Our study based on the NHANES data involving 3964 female adults suggested that higher CVH levels were associated with a shorter reproductive lifespan and a higher proportion of never-married women. ALP appeared to mediate the relationship between CVH and reproductive lifespan duration. Additionally, higher CVH levels were linked to lower odds of pregnancy loss prevalence. Promoting the convenience of data collection and calculation of LE8 score may allow the general public to get earlier practitioner-directed guidance for achieving optimal cardiovascular and reproductive health in women. Further prospective studies and mechanistic research are needed to clarify the underlying causal relationships and biological mechanisms.

## Data Availability

Publicly available datasets were analyzed in this study. This data can be found here: https://www.cdc.gov/nchs/nhanes.

## References

[1] Nabhan AF Mburu G Elshafeey F Magdi R Kamel M Elshebiny M. Women’s reproductive span: a systematic scoping review. Hum Reprod Open. (2022) 2:hoac005. doi: 10.1093/hropen/hoac005 PMC8907405 35280216

[2] Robinson GE. Pregnancy loss. Best Pract Res Clin Obstet Gynaecol. (2014) 28:169–78. doi: 10.1016/j.bpobgyn.2013.08.012 24047642

[3] Sibai B Dekker G Kupferminc M. Pre-eclampsia. Lancet. (2005) 365:785–99. doi: 10.1016/S0140-6736(05)17987-2 15733721

[4] Drenthen W Pieper PG Roos-Hesselink JW van Lottum WA Voors AA Mulder BJ. Outcome of pregnancy in women with congenital heart disease: a literature review. J Am Coll Cardiol. (2007) 49:2303–11. doi: 10.1016/j.jacc.2007.03.027 17572244

[5] Bellamy L Casas JP Hingorani AD Williams DJ. Pre-eclampsia and risk of cardiovascular disease and cancer in later life: systematic review and meta-analysis. BMJ. (2007) 335:974. doi: 10.1136/bmj.39335.385301.BE 17975258 PMC2072042

[6] Dayan N Laskin CA Spitzer K Mason J Udell JA Wald RM. Pregnancy complications in women with heart disease conceiving with fertility therapy. J Am Coll Cardiol. (2014) 64:1862–4. doi: 10.1016/j.jacc.2014.07.977 25443711

[7] Farland LV Wang YX Gaskins AJ Rich-Edwards JW Wang S Magnus MC. Infertility and risk of cardiovascular disease: A prospective cohort study. J Am Heart Assoc. (2023) 12:e027755. doi: 10.1161/JAHA.122.027755 36847044 PMC10111453

[8] Drenthen W Hoendermis ES Moons P Heida KY Roos-Hesselink JW Mulder BJ. Menstrual cycle and its disorders in women with congenital heart disease. Congenit Heart Dis. (2008) 3:277–83. doi: 10.1111/j.1747-0803.2008.00202.x 18715462

[9] Lloyd-Jones DM Allen NB Anderson CAM Black T Brewer LC Foraker RE. Life’s essential 8: updating and enhancing the american heart association’s construct of cardiovascular health: A presidential advisory from the american heart association. Circulation. (2022) 146:e18–43. doi: 10.1161/CIR.0000000000001078 PMC10503546 35766027

[10] Beroukhim G Esencan E Seifer DB. Impact of sleep patterns upon female neuroendocrinology and reproductive outcomes: a comprehensive review. Reprod Biol Endocrinol. (2022) 20:16. doi: 10.1186/s12958-022-00889-3 35042515 PMC8764829

[11] Negishi Y Shima Y Takeshita T Morita R. Harmful and beneficial effects of inflammatory response on reproduction: sterile and pathogen-associated inflammation. Immunol Med. (2021) 44:98–115. doi: 10.1080/25785826.2020.1809951 32838688

[12] Steenvoorden TS Rood JAJ Bemelman FJ Armstrong R Jr. Leuvenink HGD van der Heijden JW. Alkaline phosphatase treatment of acute kidney injury-an update. Nephrol Dial Transplant. (2024) 39:1239–47. doi: 10.1093/ndt/gfae028 PMC11334066 38400561

[13] Presbitero A Mancini E Brands R Krzhizhanovskaya VV Sloot PMA. Supplemented alkaline phosphatase supports the immune response in patients undergoing cardiac surgery: clinical and computational evidence. Front Immunol. (2018) 9:2342. doi: 10.3389/fimmu.2018.02342 30364262 PMC6193081

[14] Kerner A Avizohar O Sella R Bartha P Zinder O Markiewicz W. Association between elevated liver enzymes and C-reactive protein: possible hepatic contribution to systemic inflammation in the metabolic syndrome. Arterioscler Thromb Vasc Biol. (2005) 25:193–7. doi: 10.1161/01.ATV.0000148324.63685.6a 15499043

[15] Webber M Krishnan A Thomas NG Cheung BM. Association between serum alkaline phosphatase and C-reactive protein in the United States National Health and Nutrition Examination Survey 2005-2006. Clin Chem Lab Med. (2010) 48:167–73. doi: 10.1515/CCLM.2010.052 19958209

[16] Kim JH Lee HS Park HM Lee YJ. Serum alkaline phosphatase level is positively associated with metabolic syndrome: A nationwide population-based study. Clin Chim Acta. (2020) 500:189–94. doi: 10.1016/j.cca.2019.10.015 31678575

[17] Chen X Chen H Zhang Y Jiang Y Wang Y Huang X. Maternal liver dysfunction in early pregnancy predisposes to gestational diabetes mellitus independent of preconception overweight: A prospective cohort study. BJOG. (2022) 129:1695–703. doi: 10.1111/1471-0528.17117 35133070

[18] Wilkof-Segev R Hallak M Gabbay-Benziv R. Extremely high levels of alkaline phosphatase and pregnancy outcome: case series and review of the literature. J Perinat Med. (2021) 49:191–4. doi: 10.1515/jpm-2020-0205 32918806

[19] Ma H Wang X Xue Q Li X Liang Z Heianza Y. Cardiovascular health and life expectancy among adults in the United States. Circulation. (2023) 147:1137–46. doi: 10.1161/CIRCULATIONAHA.122.062457 PMC10165723 37036905

[20] Liu Y Tang J Gao S. The inverse relationship between Life’s Essential 8 and risk of metabolic syndrome: evidence from NHANES 2005-2018. Front Endocrinol (Lausanne). (2024) 15:1449930. doi: 10.3389/fendo.2024.1449930 39530117 PMC11551013

[21] Lin L Hu Y Lei F Huang X Zhang X Sun T. Cardiovascular health and cancer mortality: evidence from US NHANES and UK Biobank cohort studies. BMC Med. (2024) 22:368. doi: 10.1186/s12916-024-03553-2 39237921 PMC11378420

[22] Zhao Y Shi W Liu Y Qin N Huang H. Correlation between cardiometabolic index and female infertility: a cross-sectional analysis. Reprod Biol Endocrinol. (2024) 22:145. doi: 10.1186/s12958-024-01312-9 39543672 PMC11562622

[23] Guo JW Ning H Lloyd-Jones DM. Cardiovascular health status in US adults with chronic diseases: national health and nutrition examination survey (NHANES), 2013-2018. J Am Heart Assoc. (2025) 14:e034388. doi: 10.1161/JAHA.124.034388 39719405 PMC12054493

[24] Lloyd-Jones DM Ning H Labarthe D Brewer L Sharma G Rosamond W. Status of cardiovascular health in US adults and children using the american heart association’s new “Life’s essential 8” Metrics: prevalence estimates from the national health and nutrition examination survey (NHANES), 2013 through 2018. Circulation. (2022) 146:822–35. doi: 10.1161/CIRCULATIONAHA.122.060911 35766033

[25] Forman MR Mangini LD Thelus-Jean R Hayward MD. Life-course origins of the ages at menarche and menopause. Adolesc Health Med Ther. (2013) 4:1–21. doi: 10.2147/AHMT.S15946 24600293 PMC3912848

[26] Kentistou KA Kaisinger LR Stankovic S Vaudel M Mendes de Oliveira E Messina A. Understanding the genetic complexity of puberty timing across the allele frequency spectrum. Nat Genet. (2024) 56:1397–411. doi: 10.1038/s41588-024-01798-4 PMC11250262 38951643

[27] Gold EB. The timing of the age at which natural menopause occurs. Obstet Gynecol Clin North Am. (2011) 38:425–40. doi: 10.1016/j.ogc.2011.05.002 PMC3285482 21961711

[28] Taneri PE Kiefte-de Jong JC Bramer WM Daan NM Franco OH Muka T. Association of alcohol consumption with the onset of natural menopause: a systematic review and meta-analysis. Hum Reprod Update. (2016) 22:516–28. doi: 10.1093/humupd/dmw013 27278232

[29] Luo M Li J Xiao X Wu P Zhang Y. Associations between cardiovascular health and female infertility: A national population-based study. PloS One. (2024) 1:9:e0306476. doi: 10.1371/journal.pone.0306476 PMC11226045 38968246

[30] Hanson B Johnstone E Dorais J Silver B Peterson CM Hotaling J. Female infertility, infertility-associated diagnoses, and comorbidities: a review. J Assist Reprod Genet. (2017) 34:167–77. doi: 10.1007/s10815-016-0836-8 PMC5306404 27817040

[31] Liang C Chung HF Dobson AJ Cade JE Greenwood DC Hayashi K. Is there a link between infertility, miscarriage, stillbirth, and premature or early menopause? Results from pooled analyses of 9 cohort studies. Am J Obstet Gynecol. (2023) 229:47.e1– e9. doi: 10.1016/j.ajog.2023.04.009 37059411

[32] Tehrani FR Solaymani-Dodaran M Hedayati M Azizi F. Is polycystic ovary syndrome an exception for reproductive aging? Hum Reprod. (2010) 25:1775–81. doi: 10.1093/humrep/deq088 20435693

[33] Hernaez A Lee Y Page CM Skara KH Haberg SE Magnus P. Impaired glucose tolerance and cardiovascular risk factors in relation to infertility: a Mendelian randomization analysis in the Norwegian Mother, Father, and Child Cohort Study. Hum Reprod. (2024) 39:436–41. doi: 10.1093/humrep/dead234 PMC10833082 37949105

[34] Herraiz-Adillo A Higueras-Fresnillo S Ahlqvist VH Berglind D Syrjala MB Daka B. Life’s essential 8 and life’s simple 7 in relation to coronary atherosclerosis: results from the population-based SCAPIS project. Mayo Clin Proc. (2024) 99:69–80. doi: 10.1016/j.mayocp.2023.03.023 37843486

[35] Chen H Tang H Huang J Luo N Zhang X Wang X. Life’s essential 8 and mortality in US adults with chronic kidney disease. Am J Nephrol. (2023) 54:516–27. doi: 10.1159/000533257 37591229

[36] Wang MC Freaney PM Perak AM Allen NB Greenland P Grobman WA. Association of pre-pregnancy cardiovascular risk factor burden with adverse maternal and offspring outcomes. Eur J Prev Cardiol. (2022) 29:e156–e8. doi: 10.1093/eurjpc/zwab121 PMC8967477 34284496

[37] Perak AM Lancki N Kuang A Labarthe DR Allen NB Shah SH. Associations of gestational cardiovascular health with pregnancy outcomes: the Hyperglycemia and Adverse Pregnancy Outcome study. Am J Obstet Gynecol. (2021) 224:210 e1– e17. doi: 10.1016/j.ajog.2020.07.053 PMC7855033 32768430

[38] Salas-Huetos A Mitsunami M Wang S Minguez-Alarcon L Ribas-Maynou J Yeste M. Women’s adherence to healthy dietary patterns and outcomes of infertility treatment. JAMA Netw Open. (2023) 6:e2329982. doi: 10.1001/jamanetworkopen.2023 37594758 PMC10439476

[39] Wong EY Ray R Gao DL Wernli KJ Li W Fitzgibbons ED. Physical activity, physical exertion, and miscarriage risk in women textile workers in Shanghai, China. Am J Ind Med. (2010) 53:497–505. doi: 10.1002/ajim.20812 20340112 PMC2863132

[40] Tong F Wang Y Gao Q Zhao Y Zhang X Li B. The epidemiology of pregnancy loss: global burden, variable risk factors, and predictions. Hum Reprod. (2024) 39:834–48. doi: 10.1093/humrep/deae008 38308812

[41] Langley-Evans SC Pearce J Ellis S. Overweight, obesity and excessive weight gain in pregnancy as risk factors for adverse pregnancy outcomes: A narrative review. J Hum Nutr Diet. (2022) 35:250–64. doi: 10.1111/jhn.12999 PMC9311414 35239212

[42] Wang S Wang J Jiang Y Jiang W. Association between blood lipid level and embryo quality during *in vitro* fertilization. Med (Baltimore). (2020) 99:e19665. doi: 10.1097/MD.0000000000019665 PMC7220153 32221094

[43] Moley KH Chi MM Mueckler MM. Maternal hyperglycemia alters glucose transport and utilization in mouse preimplantation embryos. Am J Physiol. (1998) 275:E38–47. doi: 10.1152/ajpendo.1998.275.1.E38 9688872

[44] Basso O Rasmussen S Weinberg CR Wilcox AJ Irgens LM Skjaerven R. Trends in fetal and infant survival following preeclampsia. JAMA. (2006) 296:1357–62. doi: 10.1001/jama.296.11.1357 16985227

[45] Lu Q Zhang X Wang Y Li J Xu Y Song X. Sleep disturbances during pregnancy and adverse maternal and fetal outcomes: A systematic review and meta-analysis. Sleep Med Rev. (2021) 58:101436. doi: 10.1016/j.smrv.2021.101436 33571887

[46] Attali E Yogev Y. The impact of advanced maternal age on pregnancy outcome. Best Pract Res Clin Obstet Gynaecol. (2021) 70:2–9. doi: 10.1016/j.bpobgyn.2020.06.006 32773291

[47] Frick AP. Advanced maternal age and adverse pregnancy outcomes. Best Pract Res Clin Obstet Gynaecol. (2021) 70:92–100. doi: 10.1016/j.bpobgyn.2020.07.005 32741623

[48] Lean SC Derricott H Jones RL Heazell AEP. Advanced maternal age and adverse pregnancy outcomes: A systematic review and meta-analysis. PloS One. (2017) 12:e018 6287. doi: 10.1371/journal.pone.0186287 PMC5645107 29040334

[49] Collaborative Group on Hormonal Factors in Breast C. Menarche, menopause, and breast cancer risk: individual participant meta-analysis, including 118–964 women with breast cancer from 117 epidemiological studies. Lancet Oncol. (2012) 13:1141–51. doi: 10.1016/S1470-2045(12)70425-4 PMC3488186 23084519

[50] Katagiri R Iwasaki M Abe SK Islam MR Rahman MS Saito E. Reproductive factors and endometrial cancer risk among women. JAMA Netw Open. (2023) 6:e233 2296. doi: 10.1001/jamanetworkopen.2023.32296 PMC10481237 37669051

[51] Shuster LT Rhodes DJ Gostout BS Grossardt BR Rocca WA. Premature menopause or early menopause: long-term health consequences. Maturitas. (2010) 65:161–6. doi: 10.1016/j.maturitas.2009.08.003 PMC2815011 19733988

[52] Jiang L Hao Y Wang Y Chen Q Xin G Li P. Is early menarche related to depression? A meta-analysis. J Affect Disord. (2025) 369:508–15. doi: 10.1016/j.jad.2024.10.036 39393462

[53] Prentice P Viner RM. Pubertal timing and adult obesity and cardiometabolic risk in women and men: a systematic review and meta-analysis. Int J Obes (Lond). (2013) 37:10 36–43. doi: 10.1038/ijo.2012.177 23164700

[54] He C Zhang C Hunter DJ Hankinson SE Buck Louis GM Hediger ML. Age at menarche and risk of type 2 diabetes: results from 2 large prospective cohort studies. Am J Epidemiol. (2010) 171:334–44. doi: 10.1093/aje/kwp372 PMC2842205 20026580

[55] Fox KM Magaziner J Sherwin R Scott JC Plato CC Nevitt M. Reproductive correlates of bone mass in elderly women. Study Osteoporotic Fractures Res Group J Bone Miner Res. (1993) 8:901–8. doi: 10.1002/jbmr.5650080802 8213252

[56] Kim H Jung JH Han K Lee DY Fava M Mischoulon D. Ages at menarche and menopause, hormone therapy, and the risk of depression. Gen Hosp Psychiatry. (2023) 83:35–42. doi: 10.1016/j.genhosppsych.2023.04.001 37043925

[57] Honigberg MC Zekavat SM Aragam K Finneran P Klarin D Bhatt DL. Association of premature natural and surgical menopause with incident cardiovascular disease. JAMA. (2019) 322:2411–21. doi: 10.1001/jama.2019.19191 PMC7231649 31738818

[58] Jacobsen BK Knutsen SF Fraser GE. Age at natural menopause and total mortality and mortality from ischemic heart disease: the Adventist Health Study. J Clin Epidemiol. (1999) 52:303–7. doi: 10.1016/s0895-4356(98)00170-x 10235170

[59] Mishra GD Davies MC Hillman S Chung HF Roy S Maclaran K. Optimising health after early menopause. Lancet. (2024) 403:958–68. doi: 10.1016/S0140-6736(23)02800-3 38458215

[60] Zhang Z Nam HK Crouch S Hatch NE. Tissue nonspecific alkaline phosphatase function in bone and muscle progenitor cells: control of mitochondrial respiration and ATP production. Int J Mol Sci. (2021) 22:1140. doi: 10.3390/ijms22031140 33498907 PMC7865776

[61] Hansen GH Niels-Christiansen LL Immerdal L Nystrom BT Danielsen EM. Intestinal alkaline phosphatase: selective endocytosis from the enterocyte brush border during fat absorption. Am J Physiol Gastrointest Liver Physiol. (2007) 293:G1325–32. doi: 10.1152/ajpgi.00379.2007 17947448

[62] Balabanova L Bondarev G Seitkalieva A Son O Tekutyeva L. Insights into alkaline phosphatase anti-inflammatory mechanisms. Biomedicines. (2024) 12:2502. doi: 10.3390/biomedicines12112502 39595068 PMC11591857

[63] Simsek M Naziroglu M Simsek H Cay M Aksakal M Kumru S. Blood plasma levels of lipoperoxides, glutathione peroxidase, beta carotene, vitamin A and E in women with habitual abortion. Cell Biochem Funct. (1998) 16:227–31. doi: 10.1002/(SICI)1099-0844(1998120)16:4<227::AID-CBF787>3.0.CO;2-M 9857484

[64] Yang H Tang T Qian Q Zhang X Liu Y Zhou X. Maternal abnormal liver function in early pregnancy and spontaneous pregnancy loss: A retrospective cohort study. J Epidemiol. (2024) 35:230–6. doi: 10.2188/jea.JE20240233 PMC11979345 39581592

