# Supplementary material for: Association of cardiovascular health with reproductive lifespan and pregnancy loss: insights from NHANES 2005–2018
Source: Front Endocrinol (Lausanne). 2025 May 26;16:1597097. doi: 10.3389/fendo.2025.1597097 (PMC12146173; doi:10.3389/fendo.2025.1597097)
Supplement: Supplementary file 1 [file Table1.pdf]

Table S1 Definition and scoring approach for quantifying CVH, as per the AHA's LE8 score, and as applied in the NHANES 2005-2018

Domain	CVH metric	Method of measurement	Quantification of CVH metric - adults (≥20 years)	Quantification of CVH metric - children (up to 19 years*)
**Health Behaviors**	**Diet**	**Measurement**: Self-reported daily intake of a DASH-style eating pattern<br><br>**Example tools for measurement:** DASH diet score (populations)	Quantiles of DASH-style diet adherence<br><br>**Scoring (Population):**<br>Points Quantile<br>100 ≥95th %ile (top/ideal diet)<br>80 75th - 94th %ile<br>50 50th - 74th %ile<br>25 25th - 49th %ile<br>0 1st - 24th %ile (bottom/least ideal quartile)	Quantiles of DASH-style diet adherence; ages 2-19<br><br>**Scoring (Population):**<br>Points Quantile<br>100 ≥95th %ile (top/ideal diet)<br>80 75th - 94th %ile<br>50 50th - 74th %ile<br>25 25th - 49th %ile<br>0 1st - 24th %ile (bottom/least ideal quartile)
	**Physical activity**	**Measurement:** Self-reported minutes of moderate or vigorous physical activity per week<br><br>**Example tools for measurement:** NHANES PAQ-K questionnaire	**Metric:** Minutes of moderate (or greater) intensity activity per week<br><br>**Scoring:**<br>Points Minutes<br>100 ≥150<br>90 120 - 149<br>80 90 - 119<br>60 60 - 89<br>40 30 - 59<br>20 1 - 29<br>0 0	**Metric:** Minutes of moderate (or greater) intensity activity per week; ages 6-19<br><br>**Scoring:**<br>Points Minutes<br>100 ≥420<br>90 360 - 419<br>80 300 - 359<br>60 240 - 299<br>40 120 - 239<br>20 1 - 119<br>0 0
	**Nicotine exposure**	**Measurement:** Self-reported use of cigarettes or inhaled nicotine-delivery system	**Metric:** Combustible tobacco use or inhaled NDS use; or secondhand smoke exposure	**Metric:** Combustible tobacco use or inhaled NDS use at any age (per clinician discretion); or secondhand smoke exposure

		**Example tools for measurement:**<br>NHANES SMQ	**Scoring:**<br>Points Status<br>100 Never smoker<br>75 Former smoker, quit ≥5 y<br>50 Former smoker, quit 1 - <5 y<br>25 Former smoker, quit <1 y, or currently using inhaled NDS<br>0 Current smoker<br><br>Subtract 20 points (unless score is 0) for living with active indoor smoker in home	**Scoring:**<br>Points Status<br>100 Never tried<br>50 Tried any nicotine product, but >30 days ago<br>25 Currently using inhaled NDS<br>0 Current combustible use (any within 30 days)<br><br>Subtract 20 points (unless score is 0) for living with active indoor smoker in home
	**Sleep health**	**Measurement:** Self-reported average hours of sleep per night<br><br>**Example tools for measurement:**<br>"On average, how many hours of sleep do you get per night?" Consider objective sleep/ actigraphy data from wearable technology, if available	**Metric:** Average hours of sleep per night<br><br>**Scoring:**<br>Points Level<br>100 7 - <9<br>90 9 - <10<br>70 6 - <7<br>40 5 - <6 or ≥10<br>20 4 - <5<br>0 <4	**Metric:** Average hours of sleep per night<br><br>**Scoring:**<br>Points Level<br>100 Age-appropriate optimal range<br>90 <1 hr above optimal range<br>70 <1 hr below optimal range<br>40 1 - <2 hrs below or ≥1 hr above optimal<br>20 2 - <3 hrs below optimal range<br>0 ≥3 hrs below optimal range
**Health Factors**	**BMI**	**Measurement:** Body weight (kg) divided by height squared (m^2)<br><br>**Example tools for measurement:**<br>Objective measurement of height and weight	**Metric:** BMI (kg/m^2)<br><br>**Scoring:**<br>Points Level<br>100 <25<br>70 25.0 - 29.9<br>30 30.0 - 34.9<br>15 35.0 - 39.9<br>0 ≥40.0	**Metric:** BMI percentiles (%iles) for age and sex; ages 2-19<br><br>**Scoring:**<br>Points Level<br>100 5th - <85th %ile<br>70 85th - <95th %ile<br>30 95th %ile - <120% of the 95th %ile<br>15 120% of the 95th %ile - <140% of the 95th %ile<br>0 ≥140% of the 95th %ile

	**Blood lipids**	**Measurement:** Plasma total and HDL-cholesterol with calculation of non-HDL cholesterol<br><br>**Example tools for measurement:** Fasting or non-fasting blood sample	**Metric:** Non-HDL cholesterol (mg/dL)<br><br>**Scoring:**<br>Points Level<br>100 <130<br>60 130 - 159<br>40 160 - 189<br>20 190 - 219<br>0 ≥220<br><br>If drug-treated level, subtract 20 points	**Metric:** Non-HDL cholesterol (mg/dL); starting no later than age 9–11 y and earlier per clinician discretion<br><br>**S-coring:**<br>Points Level<br>100 <100<br>60 100 - 119<br>40 120 - 144<br>20 145 - 189<br>0 ≥190<br><br>If drug-treated level, subtract 20 points
	**Blood glucose**	**Measurement:** Fasting blood glucose or casual HbA1c<br><br>**Example tools for measurement:** Fasting (FBG, HbA1c) or non-fasting (HbA1c) blood sample	**Metric:** FBG (mg/dL) or HbA1c (%)<br><br>**Scoring:**<br>Points Level<br>100 No history of diabetes and FBG <100 (or HbA1c < 5.7)<br>60 No diabetes and FBG 100 - 125 (or HbA1c 5.7 - 6.4) (Pre-diabetes)<br>40 Diabetes with HbA1c <7.0<br>30 Diabetes with HbA1c 7.0 - 7.9<br>20 Diabetes with HbA1c 8.0 - 8.9<br>10 Diabetes with Hb A1c 9.0 - 9.9<br>0 Diabetes with HbA1c ≥10.0	**Metric:** FBG (mg/dL) or HbA1c (%), symptom-based screening at any age or risk-based screening starting at age ≥10 y or onset of puberty per clinician discretion<br><br>**Scoring:**<br>Points Level<br>100 No history of diabetes and FBG <100 (or HbA1c < 5.7)<br>60 No diabetes and FBG 100 - 125 (or HbA1c 5.7 - 6.4) (Pre-diabetes)<br>40 Diabetes with HbA1c <7.0<br>30 Diabetes with HbA1c 7.0 - 7.9<br>20 Diabetes with HbA1c 8.0 - 8.9<br>10 Diabetes with Hb A1c 9.0 - 9.9<br>0 Diabetes with HbA1c ≥10.0
	**BP**	**Measurement:** Appropriately measured systolic and diastolic BP	**Metric:** Systolic and diastolic BP (mmHg)<br><br>**Scoring:**<br>Points Level<br>100 <120/<80 (Optimal)	**Metric:** Systolic and diastolic BP (mmHg) percentiles for ages through 12 y, For age ≥13 y, use adult scoring<br><br>**Scoring:**

		**Example tools for measurement:**<br>Appropriately sized BP cuff	75 120 - 129/<80 (Elevated)<br>50 130 - 139 or 80 - 89 (Stage 1 HTN)<br>25 140 - 159 or 90 - 99<br>0 ≥160 or ≥100<br><br>Subtract 20 points if treated level	Points Level<br>100 Optimal (<90th %ile)<br>75 Elevated (≥90th - <95th %ile or ≥120/80 mmHg to <95th %ile, whichever is lower)<br>50 Stage 1 HTN (≥95th - <95th %ile + 12 mmHg, or 130/80 to 139/89 mmHg, whichever is lower)<br>25 Stage 2 HTN (≥95th %ile + 12 mmHg, or ≥140/90 mmHg, whichever is lower)<br>0 Systolic BP ≥160 or ≥95th %ile + 30 mmHg systolic BP, whichever is lower; and/or diastolic BP ≥100 or ≥95th %ile + 20 mmHg diastolic BP<br><br>Subtract 20 points if treated level

*Cannot meet these metrics until solid foods are being consumed.

Abbreviations: CVH, cardiovascular health; AHA, American Heart Association; LE8, Life's Essential 8; NHANES, National Health and Nutrition Examination Survey; DASH, Dietary Approaches to Stop Hypertension; PAQ-K, Physical Activity Questionnaire K; SMQ, smoking assessment; NDS, nicotine-delivery system; BMI, body mass index; HDL, high-density lipoprotein; FBG, fasting blood glucose; HbA1c, hemoglobin A1c; BP, blood pressure; HTN, hypertension.
