# Supplementary material for: Association of cardiovascular health with reproductive lifespan and pregnancy loss: insights from NHANES 2005–2018
Source: Front Endocrinol (Lausanne). 2025 May 26;16:1597097. doi: 10.3389/fendo.2025.1597097 (PMC12146173; doi:10.3389/fendo.2025.1597097)
Supplement: Supplementary file 2 [file Table2.docx]

**Table S2 The association between the LE8 CVH score and pregnancy loss (by zero-inflated negative binomial regression model)**

| **CVH metrics** | **Model 1**  **[β (95% CI) *P*]** | **Model 2**  **[β (95% CI) *P*]** | **Model 3**  **[β (95% CI) *P*]** |
| --- | --- | --- | --- |
| Total CVH score (per 10 scores) | -0.06 (-0.13, 0.01) 0.070 | -0.07 (-0.13, -0.02) 0.012 | -0.08 (-0.13, -0.02) 0.005 |

Model 1 was unadjusted for covariates; Model 2 enhanced Model 1 by including age and race/ethnicity; Model 3 further augmented Model 2 by integrating education level, PIR and marital status

Abbreviations: CVH, cardiovascular health; LE8, Life’s Essential 8
